# The Dark Proteome Database

**DOI:** 10.1186/s13040-017-0144-6

**Published:** 2017-07-20

**Authors:** Nelson Perdigão, Agostinho C. Rosa, Seán I. O’Donoghue

**Affiliations:** 1 Instituto Superior Técnico, Universidade de Lisboa, 1049-001 Lisbon, Portugal; 2 Instituto de Sistemas e Robótica, 1049-001 Lisbon, Portugal; 3 Garvan Institute of Medical Research, Sydney, NSW 2010 Australia; 4 The University of Sydney, Sydney, NSW 2006 Australia; 5 Commonwealth Scientific and Industrial Research Organisation (CSIRO), Sydney, NSW 1670 Australia

**Keywords:** Dark Proteome, Molecular Structure, Homology Modelling

## Abstract

**Background:**

Recently we surveyed the dark-proteome, i.e., regions of proteins never observed by experimental structure determination and inaccessible to homology modelling. Surprisingly, we found that most of the dark proteome could not be accounted for by conventional explanations (e.g., intrinsic disorder, transmembrane domains, and compositional bias), and that nearly half of the dark proteome comprised dark proteins, in which the entire sequence lacked similarity to any known structure. In this paper we will present the Dark Proteome Database (DPD) and associated web services that provide access to updated information about the dark proteome.

**Results:**

We assembled DPD from several external web resources (primarily Aquaria and Swiss-Prot) and stored it in a relational database currently containing ~10 million entries and occupying ~2 GBytes of disk space. This database comprises two key tables: one giving information on the ‘darkness’ of each protein, and a second table that breaks each protein into dark and non-dark regions. In addition, a second version of the database is created using also information from the Protein Model Portal (PMP) to determine darkness. To provide access to DPD, a web server has been implemented giving access to all underlying data, as well as providing access to functional analyses derived from these data.

**Conclusions:**

Availability of this database and its web service will help focus future structural and computational biology efforts to study the dark proteome, thus providing a basis for understanding a wide variety of biological functions that currently remain unknown.

**Availability and implementation:**

DPD is available at http://darkproteome.ws. The complete database is also available upon request. Data use is permitted via the Creative Commons Attribution-NonCommercial International license (http://creativecommons.org/licenses/by-nc/4.0/).

**Electronic supplementary material:**

The online version of this article (doi:10.1186/s13040-017-0144-6) contains supplementary material, which is available to authorized users.

## Background

Two key databases in protein biochemistry are UniProt [1], which records protein sequences, and the Protein Data Bank (PDB) [2], which records three-dimensional (3D) structural models derived from experiments such as X-ray crystallography. Comparing these two databases in terms of number of entries, UniProt has more than 65 million sequences while PDB has only ~125,000 3D structures in 2017. Since multiple PDB entries are often derived for the same protein, the number of unique protein sequences in PDB is even less (~40,000); for the human organism PDB holds ~34,500 structures. This means that only <0.1% of protein sequences in UniProt have an experimentally determined 3D structure. Nevertheless about half of all protein sequences are detectably similar to proteins with known 3D structure, and therefore some three-dimensional (3D) structural information can be inferred by homology modelling [3, 4].

However, many life scientists fail to benefit from structure information prevenient of homology modelling because is often difficult to access and use. Out of this need was born SRS 3D, a module of SRS [5], and its more recent successor Aquaria [6] - two services designed to make 3D homology model information more readily accessible. Aquaria is derived by systematically comparing all PDB proteins against 546,000 Swiss-Prot sequences [1], which essentially covers all well-described protein sequences across a wide range of organisms. This comparison resulted in 46 million sequence-to-structure alignments on PSSH2 database [6] resulting in one matching structure, at least, for 87% of Swiss-Prot proteins and a median of 35 structures per protein, therefore providing a depth of sequence-to-structure information currently not available from other resources in nowadays.

Recently, we used Aquaria’s set of 46 million sequence-to-structure alignments to examine the ‘dark’ proteome, i.e., the protein sequences (full or partial) that are not detectably similar in sequence to any sequence with known structure in PDB [6]. By “dark” we mean labelled as unknown, knowing that some structure is present like in dark matter (metaphorically) independent of their nature. Some are using the term “dark” proteome as a synonym of Intrinsically Disordered Proteins (IDP’s) [7] but this definition is highly incomplete because we surprisingly found that much of the dark proteome contain ‘unknown unknowns’ regions, i.e., regions that cannot be easily explained by factors such as disorder or transmembrane domains, in fact more than half of the dark proteome in the four domains of life is ordered, globular and with low compositional bias [8]. In addition, we found also that around 15% of the all Swiss-Prot is composed of dark proteins i.e., proteins where the entire (100% of the) sequence is not detectably similar to anything in PDB. Half of these dark proteins showed unexpected features like the ones described above (ordered, globular, and low compositional bias) [8].

In this work we announce the Dark Proteome Database (DPD), an online and updated version of the database used in our previous work [8] indicating whenever possible the nature of the dark and non-dark regions (disordered, transmembrane, and compositional bias) among other features for each Swiss-Prot protein. In this work we also indicate predictions for disordered and transmembrane regions using PredictProtein (PP) [9]. By making this data broadly accessible to life scientists, this work will help shed light on the remaining dark proteome of structural biology.

## Implementation

### Database

**Fig. 1:**
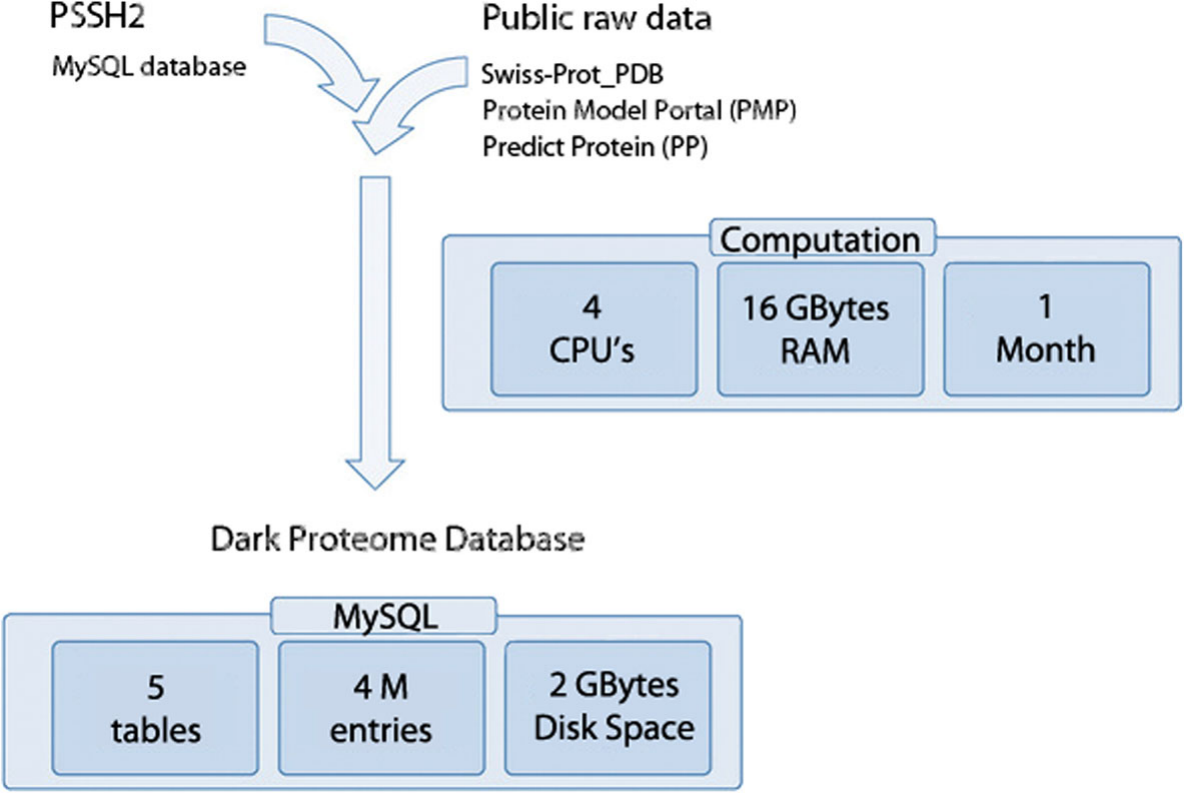
Flux of data into Dark Proteome Database. Visualization showing how the DPD is built

**Fig. 2:**
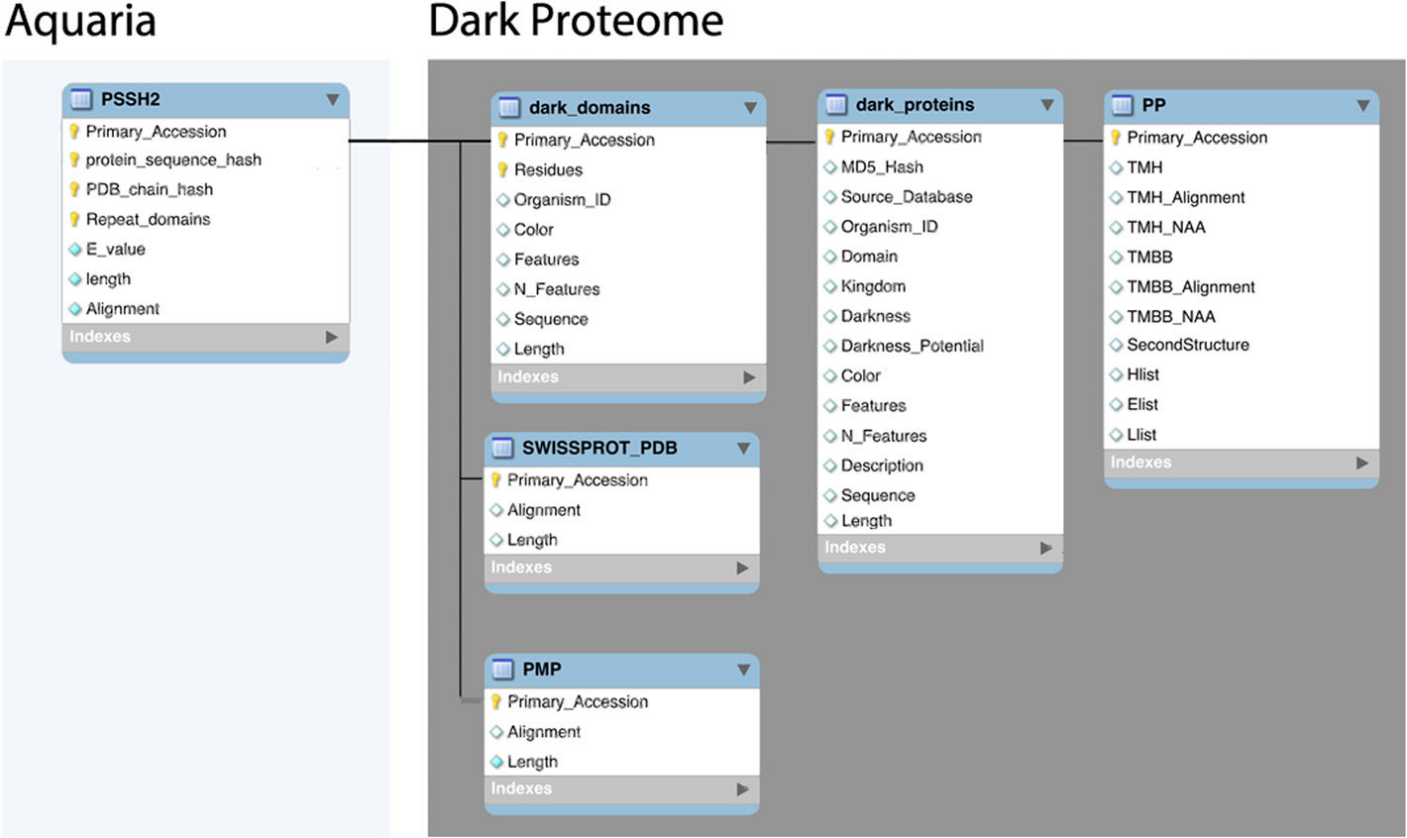
Aquaria and Dark Proteome database relational schema. Schema showing how the two databases are related

DPD is created by a pipeline (Fig. 1) that brings together information from PSSH2 (‘Protein Sequence-to-Structure Homologies’) - the database underlying Aquaria [6], Swiss-Prot [1], and the Protein Model Portal (PMP) [4]. In the DPD pipeline, the following three initial steps are used to map the dark regions for each protein sequence present in Swiss-Prot (Fig. 1):

1. The first step concerns all sequence-to-structure alignments available in PSSH2. The complete Aquaria entry for each protein is fetched (e.g., Q9H324). This entry is then analysed to determine which amino acid residues are not matched to any homologous PDB structure.
2. The second step concerns reliability by getting the possible alignment between a Swiss-Prot sequence and its PDB homologues using UniProt Consortium criteria instead of PSSH2. This alignment information can be downloaded from the following URL http://www.uniprot.org/uniprot/?query=database%3A%28type%3Apdb%29&sort=score. The file content is converted into a MySQL table afterwards denominated SWISSPROT_PDB that is part of the Dark Proteome database (Fig. 2). By doing this we don’t lose alignments that exists de facto, but that were missed by PSSH2 detection algorithm (HHblits [10]).
3. The third step regards completeness by fetching the corresponding predicted PMP entry (e.g. http://www.proteinmodelportal.org/query/up/Q9H324) and uses it to identify regions that contain sequence-to-structure alignments missed by both HHblits and UniProt.

**Fig. 3:**
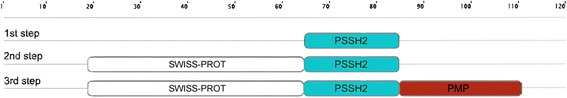
Three steps domains fulfilment. Example shown for human (organism ID number 9606) protein Q9H324

**Fig. 4:**
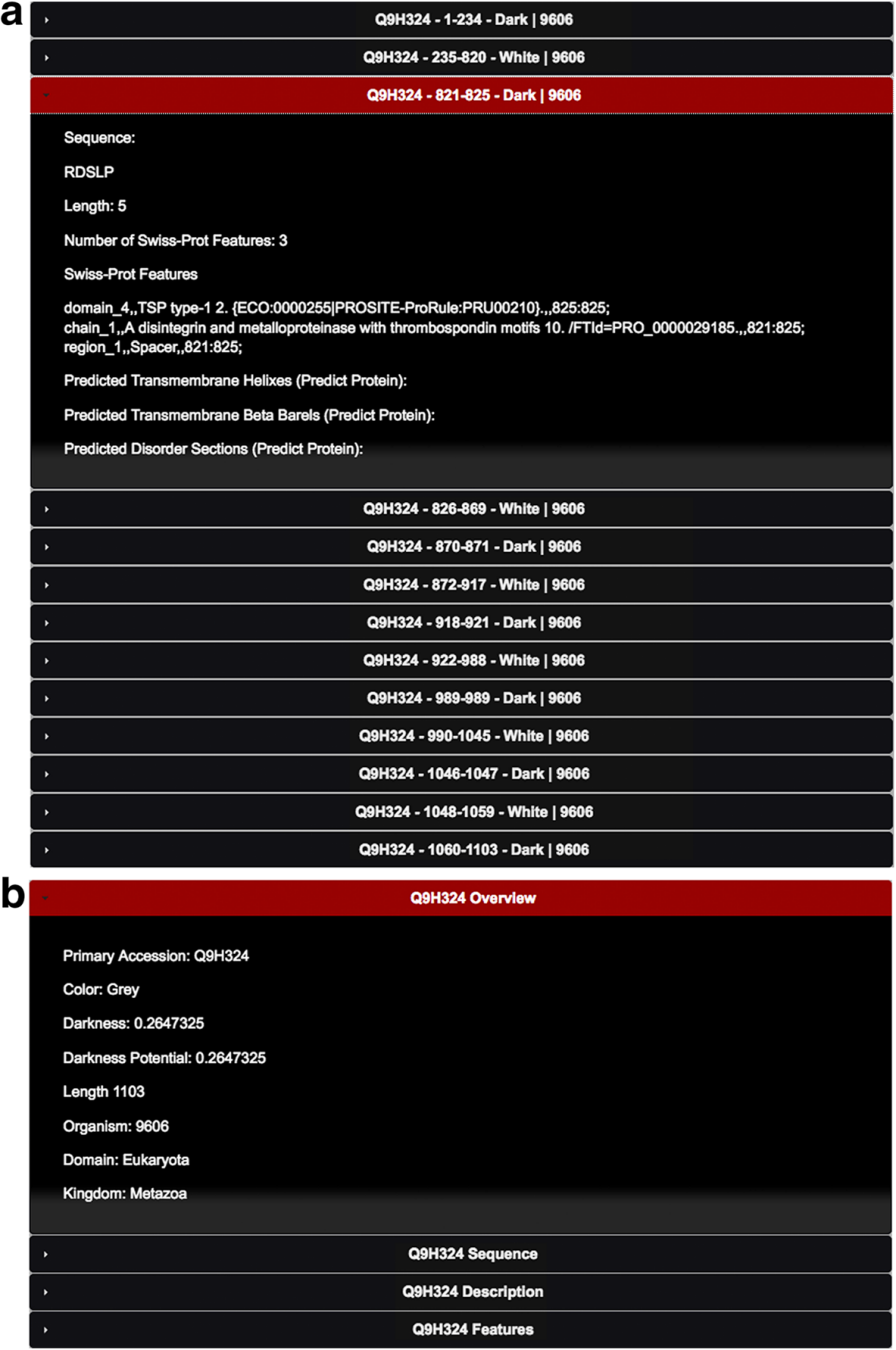
Web interface with PMP. **a** dark_domains table holding colour domains for protein Q9H324 through DPD. **b** dark_proteins table showing entry for protein Q9H324 holding colour Grey for the full protein

The above information is then used to assemble a MySQL table called ‘dark_domains’ (Fig. 2). Each entry in this table corresponds to a ‘white’ or ‘dark’ region of a protein, defined as follows:

- White regions indicate a contiguous region of the amino acid sequence in which all (100% of the) residues are aligned to a 3D structure in either Swiss-Prot, PSSH2, or PMP (Figs. 3 and 4a);
- Dark regions are contiguous regions of the amino acid sequence in which no (0% of the) residues are aligned to a structure in the above step (Figs. 3 and 4a).

Next, we use the dark_domains table to create a second table called ‘dark_proteins’ (Fig. 4b). Each entry in this table corresponds to a protein, which is assigned to be either ‘White’, ‘Dark’, or ‘Grey’ as follows (Fig. 4b):

- White, if and only if the entire (100% of the) amino acid sequence of the protein is one single white domain;
- Dark, if and only if the entire (100% of the) amino acid sequence of the protein is one single dark domain;
- Grey, if the protein contains both dark and white domains.

**Fig. 5:**
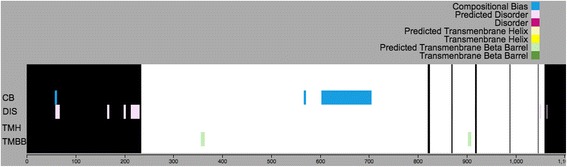
Dark Proteome map with PMP for protein Q9H324

Predictions from PredictProtein (PP) [9] are inserted into PP table in the Dark Proteome Database (Figs. 2 and 5).

**Fig. 6:**
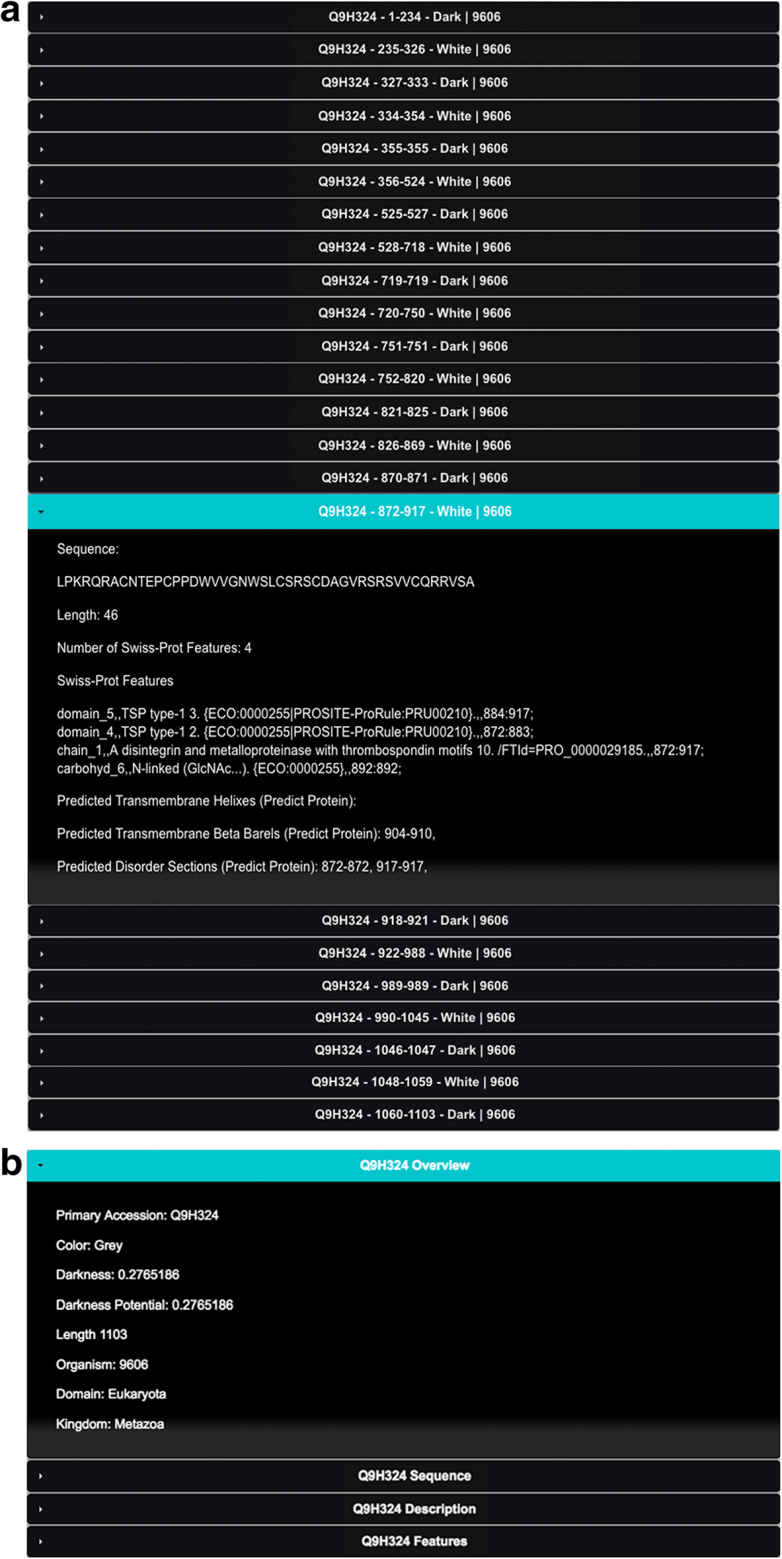
Web interface without PMP. **a** Dark_domains table holding colour domains for protein Q9H324, where PMP regions are ignored, i.e., they are considered dark. **b** dark proteins table showing entry for protein Q9H324 holding colour Grey for the full protein

For last we create a second version of the DPD that uses only Aquaria and UniProt data. In this version, the ‘dark_domains’ table is generated as follows (Fig. 6a):

- White regions indicate a contiguous region of the amino acid sequence in which all (100% of the) residues are aligned to a 3D structure in either PSSH2 or UniProt;
- Dark regions are contiguous regions of the amino acid sequence in which no (0% of the) residues are aligned to a structure in the above step.

Similarly, a second ‘dark_proteins’ table (Fig. 6b) is generated based on the previous ‘dark_domains’ table (Fig. 6a).

**Fig. 7:**
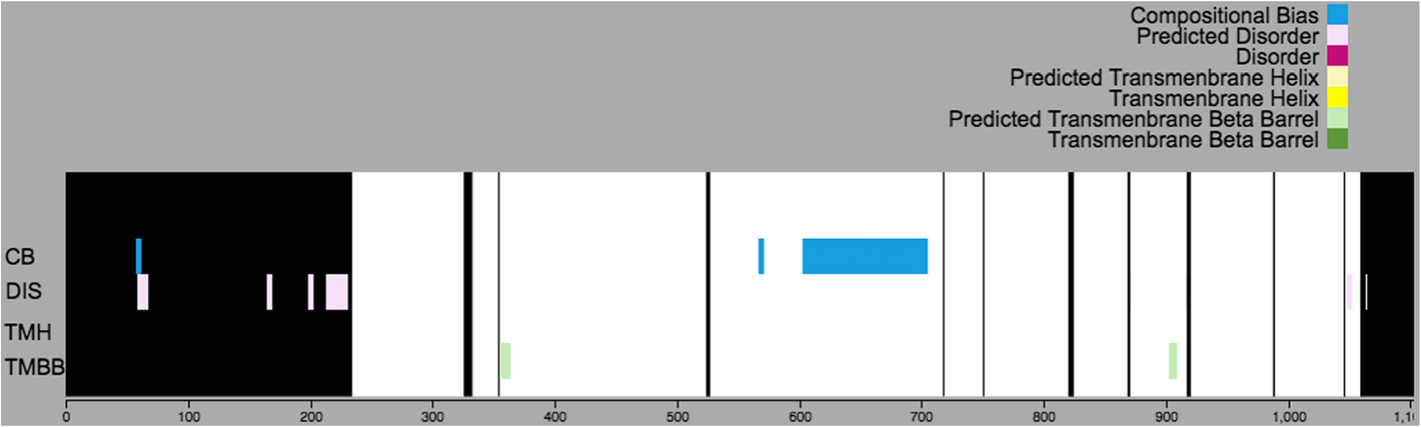
Dark Proteome map without PMP for protein Q9H324

Likewise predictions from PredictProtein (PP) [9] are inserted into PP table in the Dark Proteome Database (Figs. 2 and 7).

The overall process of fetching and assembling data takes around one month using a Quad-core i7 at 2.8 GHz; however, as most of these source services have multicore servers, the process can be speed up by parallel data fetching.

The current version of DPD (July 2016) was assembled using PSSH2, Swiss-Prot, and PMP from July 2016. The complete database contains around 10 million entries (including entries both with and without PMP) and occupies around 2Gb in disk space. [Each time the database is updated, we immediately run a series of validation tests that check overall features, as well as a range of specific smoke cases for individual proteins (Additional file 1)].

### Web service

The DPD web service is built using Apache, PHP, MySQL, JQuery, JQueryUI, and d3.js. On the DPD homepage a client-side AJAX engine initiates HTTP GET requests to the server, sending user-selected options. The AJAX engine notifies the user that a search has been initiated by displaying an animated ‘throbber’ icon. After the server-side PHP script receives the search options from the GET request, it constructs and executes the appropriate MySQL query on the database. Once the query has been executed, the script builds a JSON object from the result set and returns it to the AJAX engine. Upon receiving the JSON response, the AJAX engine parses it, builds the mark-up for the results and displays it in the browser window.

## Results

The database is web-accessible allowing fast access to any Swiss-Prot protein information, revealing either the dark and non-dark regions and their corresponding characterization (disordered, transmembrane, and compositional bias). Predictions from PredictProtein are also available for disorder and transmembrane regions. The user can choose to see data from either version of the database, thus enabling them to use a definition of darkness that either excludes or includes PMP. The web interface therefore provides users with a fast access to individual entries for any protein, revealing either the dark and non-dark regions (e.g., http://darkproteome.ws/database/domains.php?id=Q9H324) (Fig. 4a), or the overall percentage of dark residues (e.g., http://darkproteome.ws/database/protein.php?id=Q9H324) (Fig. 4b).

**Fig. 8:**
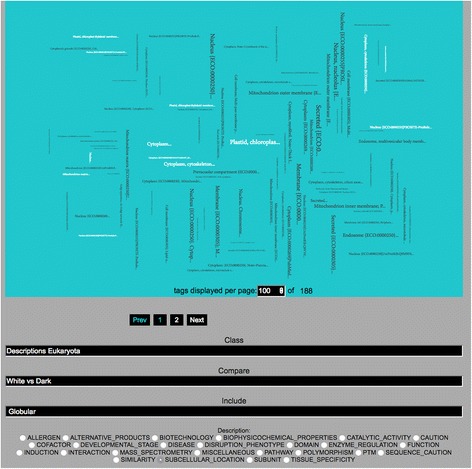
Tag-cloud with pagination. Visualization showing subcellular locations over- or under-represented in dark eukaryotic proteins (dark and white text, respectively). Text size terms in the tag cloud is set to the minus log of significance (score computed by adjusted Fisher’s exact test). Annotations are sorted into categories and pages, helping thus making this very large set of annotations more manageable. This tool provides insight into a very wide variety of biological questions

**Fig. 9:**
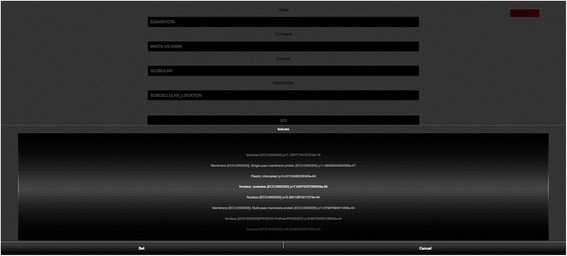
Spinning wheels. Visualization showing subcellular locations over- or under-represented in dark eukaryotic proteins). The order of terms in the spinning wheel is set to the minus log of significance (score computed by adjusted Fisher’s exact test). This tool therefore also provides insight into a very wide variety of biological questions

We also provide some functional analyses, by comparing annotations between dark and non-dark sets in a reliable manner, where we applied annotation enrichment for the ‘Description’ field of the Swiss-Prot proteins through Fisher exact tests [11, 12] with adjustment [8, 13]. The analyses results are presented in a Tag Cloud with pagination [14] to reveal the most functional terms over- or under-represented in dark proteins or dark regions (Fig. 8). Spinning wheels are also available and can be seen as an alternative visualization method to the tag cloud, where the results are revealed as sorted lists (Fig. 9).

## Discussion

We have already observed that specific examination of the dark proteome led to some surprising results and has challenged some of the current beliefs and conceptions, in what concerns the proteome that remain inaccessible to structural biology determination or modelling [8]. We stress again that more than half of the dark proteome is ordered, globular and with low compositional bias, with this work we want also to reinforce the difference between darkness and intrinsic disorder proteins (IDP’s). Some researchers renamed IDP’s as ‘dark’ proteome [7] but under our perspective this is incomplete. Our definition of ‘dark’ is more broad and consistent. We proved that IDP’s don’t explain not even one fourth of the dark proteome [8].

Having this in mind, DPD was designed considering two usage scenarios: Protein based - when a researcher wants to know about how much of a Swiss-Prot protein is known (by inserting the Swiss-Prot ID in the site header) i.e., protein dark and white domains and its characterization in terms of disorder, transmembrane, or compositional bias using Swiss-Prot annotations and/or predictions from different sources like Predict Protein or PMP; Domain based – when a researcher wants to overview which descriptions stand out for the four domains of life through Fisher tests [11–13] visualizing them by tag clouds and/or spinning wheels (available through the Fisher tests menu).

Therefore, this database is a general map for the dark proteome, at the present time, and where organism’s information will be available in the future (a third usage scenario) together with its respective functional analysis so that an organism could be more understandable (the Human organism is already present in the tag cloud and in the spinning wheels for instance, where other organisms will follow).

From the last paragraph, we can clearly understand that DPD is a work in progress where it’s intended to extend this database in two main axis: the first concerns the domain of coverage where we are already working with TrEMBL [1]; the second is related with the inclusion of new sources of annotations and predictions. By doing this, DPD certainly will become an essential and reliable tool, like it was in the past [8], to map and describe the *ignota mundi* of the dark proteome.

## Conclusion

We believe there are many further discoveries waiting to be made by further studying these regions and exploring the role of the dark proteome in specific biological functions or in human health, specifically the many proteins that are part of the dark proteome and are involved in various different functions in the cell, like cellular signalling or cellular organization; undoubtedly, many of these proteins will be associated with diseases, such as cardiovascular disease, cancer, diabetes, neurodegenerative diseases such as Parkinson or Alzheimer. This work therefore, will consolidate structural knowledge from Aquaria, UniProt, PDB and PMP into an easy-to-use interface that gives users quick access to the precise mapping of dark and non-dark regions, domains of life and organisms (in a near future). Thus, DPD will help focus further research while shedding light on the remaining dark proteome and revealing molecular processes of life that are currently unknown.

## Additional files

Additional file 1: An image file showing the validation tests used to check overall features and a range of individual proteins. (ZIP 1123 kb)

## Abbreviations

DPD: Dark Proteome Database
PMP: Protein Model Portal
PSSH2: Protein Sequence-to-Structure Homologies
