# Supplementary figures and images for: The Dark Proteome Database
Source: BioData Min. 2017 Jul 20;10:24. doi: 10.1186/s13040-017-0144-6 (PMC5520327; doi:10.1186/s13040-017-0144-6)

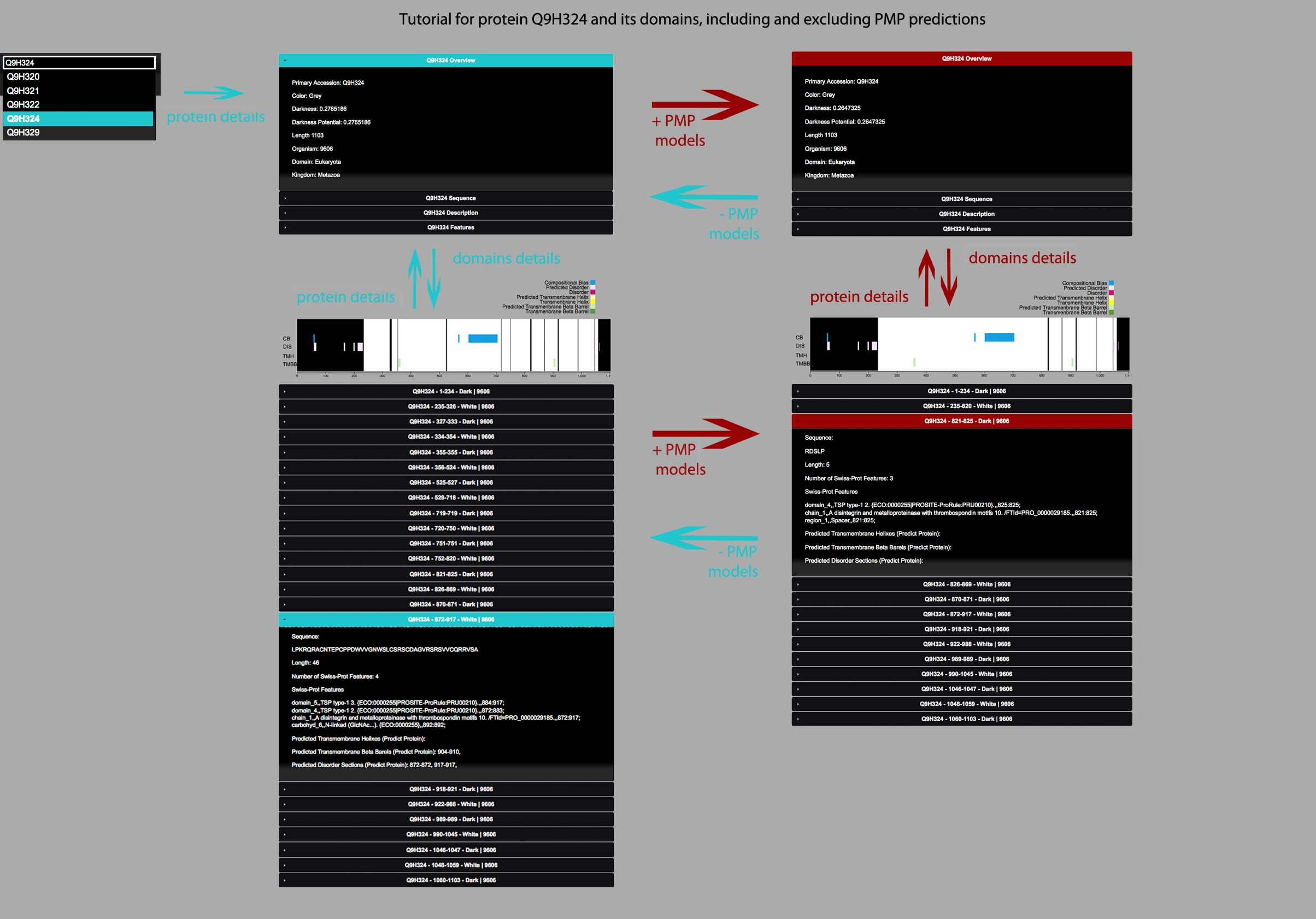

Supplement: Supplementary file 1 — An image file showing the validation tests used to check overall features and a range of individual proteins. (ZIP 1123 kb) [file 13040_2017_144_MOESM1_ESM.zip › SF3R2.tif]

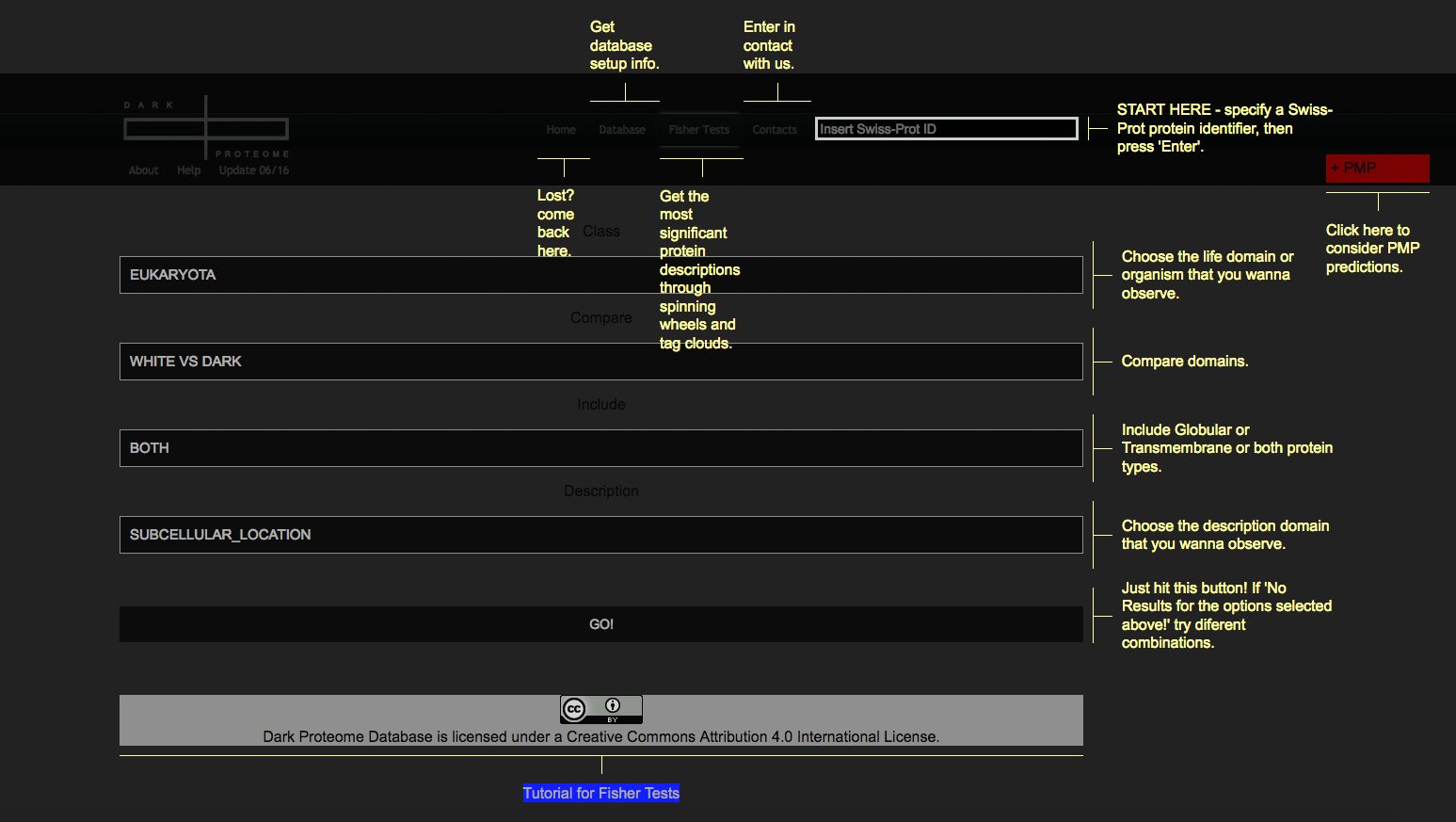

Supplement: Supplementary file 1 — An image file showing the validation tests used to check overall features and a range of individual proteins. (ZIP 1123 kb) [file 13040_2017_144_MOESM1_ESM.zip › SF2R2.tif]

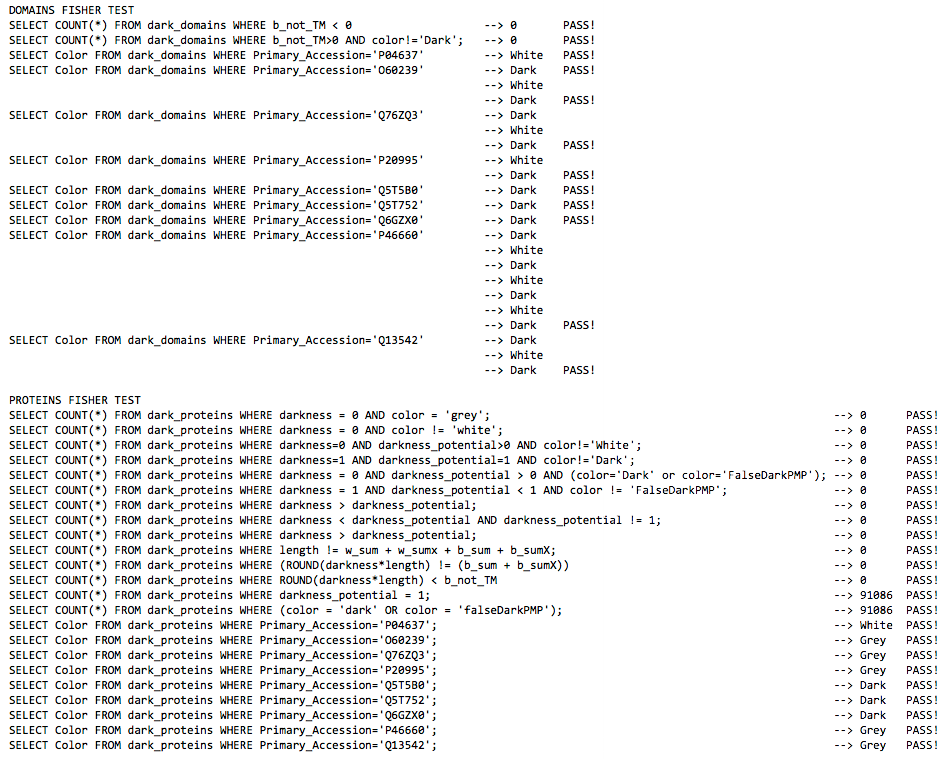

Supplement: Supplementary file 1 — An image file showing the validation tests used to check overall features and a range of individual proteins. (ZIP 1123 kb) [file 13040_2017_144_MOESM1_ESM.zip › SF1R2.tif]
